# Adding climate change to the mix: responses of aquatic ectotherms to the combined effects of eutrophication and warming

**DOI:** 10.1098/rsbl.2021.0442

**Published:** 2021-10-27

**Authors:** Essie M. Rodgers

**Affiliations:** School of Biological Sciences, University of Canterbury, Private Bag 4800, Christchurch 8140, New Zealand

**Keywords:** nutrients, climate warming, global change, eutrophication, algal blooms, fish

## Abstract

The threat of excessive nutrient enrichment, or eutrophication, is intensifying across the globe as climate change progresses, presenting a major management challenge. Alterations in precipitation patterns and increases in temperature are increasing nutrient loadings in aquatic habitats and creating conditions that promote the proliferation of cyanobacterial blooms. The exacerbating effects of climate warming on eutrophication are well established, but we lack an in-depth understanding of how aquatic ectotherms respond to eutrophication and warming in tandem. Here, I provide a brief overview and critique of studies exploring the cumulative impacts of eutrophication and warming on aquatic ectotherms, and provide forward direction using mechanistically focused, multi-threat experiments to disentangle complex interactions. Evidence to date suggests that rapid warming will exacerbate the negative effects of eutrophication on aquatic ectotherms, but gradual warming will induce physiological remodelling that provides protection against nutrients and hypoxia. Moving forward, research will benefit from a greater focus on unveiling *cause and effect* mechanisms behind interactions and designing treatments that better mimic threat dynamics in nature. This approach will enable robust predictions of species responses to ongoing eutrophication and climate warming and enable the integration of climate warming into eutrophication management policies.

## 1. Introduction

Anthropogenic eutrophication (hereafter, eutrophication) is the world's most widespread form of habitat degradation affecting aquatic ecosystems [1,2]. Excessive nutrient inputs trigger eutrophication events where rapid, uncontrolled growth of aquatic plants is spurred and harmful algal blooms spread [3,4]. Phosphorus (P) and nitrogen (N) are the key nutrients of concern because their availability drives aquatic plant primary production. In freshwater habitats, plant growth is limited by P availability, whereas N is generally the limiting nutrient in marine habitats [5,6]. Aquatic habitats transform during eutrophication; floating plants and cyanobacteria become over-abundant and dominant over other plant life, creating low light conditions for underwater life and nightly hypoxic (low oxygen) episodes [7]. Water quality also declines, with increased turbidity levels and high concentrations of dissolved nutrients, many of which can disrupt homeostasis in aquatic ectotherms by passively diffusing across the gills and epithelium [3,8,9]. Following eventual bloom die-off, bacterial decomposition consumes large amounts of oxygen and produces carbon dioxide causing hypoxia and acidification, respectively [10]. The degraded habitat conditions driven by eutrophication have been linked to mass mortalities of aquatic life the world over [4,11–13], and this loss of life is becoming more common as habitats warm [14].

The environmental consequences and economic burden of eutrophication are predicted to surge under forecasted climate change [15,16]. In 2009, the annual economic cost of eutrophication (e.g. lost tourism revenue and commercial fisheries) was estimated to be £114 million in England and Wales [17], and USD$2.2 billion in the United States (US) [18], but these estimates have not factored in the catalysing effects of climate change [15]. Heatwaves are increasing in intensity, frequency and duration around the world [14]. For example, the 2018 European heatwave lasted several weeks and had devastating impacts on aquatic life [19]; over 5 t of dead fish were found in the Rhine, Elbe and other rivers in Germany when water temperature increased 4°C above summertime normal. The heatwave was linked to the formation of one of the largest algal blooms in the Baltic Sea and a ‘dead zone', with insufficient oxygen to support life, spanning 70 km^2^. Here, I provide an overview of how aquatic ectotherms are affected by eutrophication and climate warming in tandem and highlight knowledge gaps to direct further research.

## 2. Climate change catalyses eutrophication

Climate change catalyses eutrophication by creating conditions that increase nutrient loadings in aquatic habitats and support rapid algal growth [15,16,20]. Elevated temperatures can indirectly increase the release of nutrients from lake sediment and catchment soils, promoting more rapid algal growth [21]. Additionally, cyanobacteria typically grow more efficiently at high temperatures compared to other phytoplankton species, suggesting it will have a competitive advantage under future warming [22,23]. Heat-accelerated growth of cyanobacteria suggests that blooms will form faster, earlier in the year and reach larger sizes with climate warming. More expansive algal blooms may cause more severe hypoxic episodes, which occur nightly when plants cannot photosynthesize and during the eventual die-off and microbial decomposition of blooms [12].

Altered precipitation patterns can also exacerbate eutrophication [24]. Increases in storm frequency and severity are projected to result in greater groundwater and surface nutrient discharge into freshwater and coastal habitats. In the United States, for example, total N loadings in riverine habitats are predicted to increase by approximately 19% by 2100 due to changes in precipitation patterns, and similar increases are expected to occur in India, China and Southeast Asia [24]. More frequent drought periods can also increase nutrient loadings by reducing habitat water levels and increasing the dissolved concentrations of nutrients [21]. Recent evidence suggests that eutrophication, in itself, may be contributing to climate change by lowering the carbon sequestration rates of seagrass beds [25], and releasing nitrous oxide and methane into the atmosphere [26,27]. Evidence supporting the strengthening of eutrophication under climate warming and the positive feedback loops between these threats is strong, but how aquatic organisms will respond to both threats in concert remains less clear.

## 3. Eutrophication: a cocktail of interacting threats

**Figure 1.**
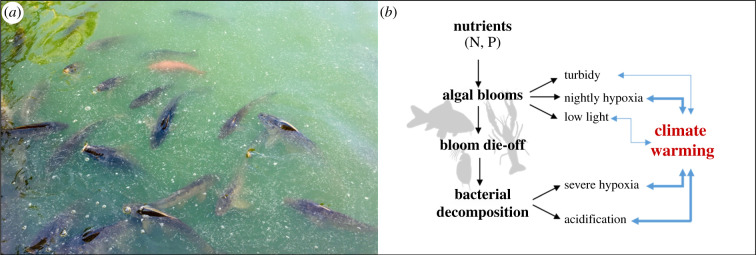
(*a*) Fish inhabiting a eutrophic lake in Seville, Spain (photo credit: Daniel Gomez Isaza). (*b*) Diagram showing the sequence (black arrows) of threats that aquatic ectotherms face during eutrophication, with the added threat of climate warming. Some threats are experienced sequentially, while others are experienced simultaneously. Excessive nutrient enrichment (nitrogen [N] in marine habitats, phosphorus [P] in freshwater habitats) leads to accelerated algal and cyanobacteria bloom growth, causing increased turbidity, nightly hypoxia and low light levels. When blooms die, bacterial decomposition of the plant matter consumes oxygen and produces carbon dioxide, causing hypoxia and acidification, respectively. Blue arrows represent the interactions between climate warming and eutrophication threats and arrow thickness reflects the extent of literature on these interactions (thicker arrows reflect more literature than thinner arrows). Multi-way threat interactions are not shown, but much less literature exists for these compared to two-way interactions.

Although eutrophication is often referred to as a single threat (or ‘stressor'), the process of eutrophication exposes organisms to complex combinations of challenges, including elevated nutrient concentrations, harmful algal blooms, increased turbidity, low light levels, hypoxic conditions, pH reductions and altered plant and animal communities (figure 1). Many of these threats are experienced sequentially (e.g. nutrient exposure and subsequent hypoxia), but most multi-threat studies have exposed organisms to threats simultaneously. To understand how organisms cope with eutrophication, threat interactions must be characterized in an ecologically relevant manner, alongside assessments of how climate warming may compound or alleviate these interactions. Investigations into the interactions among eutrophication threats and climate warming are still in their infancy, but simultaneous exposure to these threats may have dire consequences for aquatic ectotherms.

Nutrient loadings are a persistent background threat in eutrophic habitats, because animals must contend with regular influxes (e.g. nitrate, nitrite, ammonia and phosphorus) from wastewater discharges, aquaculture operations and run-off from urban, agricultural and mining sources [3]. Nitrate is the most stable and abundant form of nitrogen in aquatic habitats [3], and most eutrophication studies have, therefore, focused on nitrate effects. Chronic nitrate exposure can exert a range of lethal and sub-lethal effects on aquatically respiring species. A recent meta-analysis, based on data from 68 studies on freshwater fish, amphipods and amphibians, showed that long-term exposure to nitrate pollution reduced activity levels by 79%, growth by 29% and survival by 68% [28]. Moreover, the effects of nitrate pollution were shown to be worsened by the presence of additional threats, such as hypoxia and low pH, which are common threats in eutrophic habitats [10,28].

Recent evidence is mounting to show that chronic nitrate exposure can make fish more susceptible to hypoxia. For example, silver perch (*Bidyanus bidyanus*) exposed to nitrate pollution (50 or 100 NO_3_^−^ mg l^−1^) for three weeks suffered reduced hypoxia tolerance [29]. Similar findings have also been reported in a freshwater salmonid (*Thymallus thymallus*), where hypoxia tolerance decreased by 15% in fish exposed to nitrate (50 or 200 NO_3_^−^ mg l^−1^) for eight weeks, compared to controls (0 NO_3_^−^ mg l^−1^) [30]. Heightened hypoxia susceptibility in nitrate-exposed fish is linked to the toxic action of nitrate (and nitrite). Once nitrite enters the body of fish via the gills, it directly lowers blood oxygen-carrying capacity by oxidizing haemoglobin to a non-oxygen binding form, called methaemoglobin [9]. This reduction in blood oxygen-carrying capacity can scale up to reduce aerobic scope (maximum resting metabolic rate) [31–33], which is a measure of the oxygen available to support aerobic activities like locomotion and digestion. Compared to freshwater fishes, marine and estuarine fishes are more tolerant of nitrate/nitrite because chloride in sea/brackish water competitively inhibits nitrate/nitrite uptake across the gills [9].

## 4. Heatwave and warming impacts

Mass mortalities of aquatic ectotherms, or fish kills, during summer heatwaves are becoming a new norm, and are often associated with eutrophic conditions [14,34–36]. Understanding how eutrophication interacts with elevated temperatures is, therefore, key to preventing further loss of aquatic life. Elevated temperatures have profound, direct impacts on the physiology and fitness of aquatic ectotherms due to the tight relationship between environmental temperature and body temperature. Rapid increases in environmental temperature can raise the ‘cost of living' in ectotherms by increasing resting metabolic rates in an exponential manner [37]. If resting metabolic rates increase without a matched increase in maximum metabolic rates, ectotherms suffer from reduced aerobic scope, and a reduced capacity to perform aerobically supported activities [38]. Gradual increases in mean habitat temperatures allow time for thermal acclimatization responses in ectotherms, where underlying physiology is remodelled so that performance is maintained at elevated temperatures [39]. By contrast, heatwaves involve rapid spikes in environmental temperature, often leaving ectotherms insufficient time for acclimatization. Interactions between eutrophication processes and gradual increases in habitat temperatures may, therefore, be distinct to interactions with heatwaves.

Nutrient exposure can reduce aerobic scope in fish [33,40], but this effect reveals an ecological surprise when fish are acclimated to elevated temperatures. For example, five to eight weeks of acclimation to elevated temperatures offset the negative effects of nitrate on aerobic scope in silver perch [40], and caused synergistic increases in aerobic scope in both European grayling and common carp (*Cyprinus carpio*) [41,42]. These protective benefits were attributed to thermal acclimation responses, involving changes to oxygen supply and delivery systems. Thermal acclimation also confers increased tolerance to hypoxia in many fish and aquatic invertebrates [43]. Both Arctic charr (*Salvelinus alpinus*) and landlocked salmon (*Salmo salar m. sebago*) showed improved hypoxia tolerance (22–200% improvement) when fish were acclimated to high temperatures and nightly hypoxia together, and this improvement was related to remodelling of cardiac tissue [44]. By contrast, acute increases in temperature typically decrease hypoxia tolerance in aquatic ectotherms, because there is insufficient time for thermal acclimation to take place [43]. Taken together, these findings suggest that gradual habitat warming, where thermal acclimatization can occur, will enhance species resilience to nutrients and hypoxia, but rapid temperature spikes will exacerbate these threats.

For gilled-organisms like fish, tadpoles and crustaceans, heatwaves pose the added threat of increasing the uptake and accumulation of unwanted substances via increased respiration rates [45]. Dissolved nutrients, suspended sediments and contaminants can enter their gills at an increased rate as temperatures rise, but disentangling these interactions can be challenging because chemical availability and organismal detoxification mechanisms are also temperature-dependent [45]. Moreover, fish exposed to nutrients (nitrite or nitrate) generally suffer reduced heat tolerance compared to unexposed fish [30,46]. Specifically, the upper thermal limit of common carp was reduced by 1.2°C following 7 days of nitrite exposure (1 mM) [46], and dropped by 1°C in European grayling following eight weeks of exposure to nitrate (50 or 100 NO_3_^−^ mg l^−1^) [30]. Nitrate and phosphate exposure can also compromise the resilience of corals to warming [47], and lower coral bleaching thresholds [48]. Limiting nutrient loads in habitats may, therefore, have the added benefit of increasing species resilience to heatwaves.

At the population level, dire effects have been observed in fish when warming and nutrients are combined under experimental conditions. For example, the interactive effects of climate warming (+4°C) and nutrient loadings (250–2500 µg l^−1^ N; 50 µg l^−1^ P) were examined in three-spined stickleback (*Gasterosteus aculeatus*) populations, using a full-factorial design with 24 freshwater mesocosms across 16 months [49]. Stickleback populations became extinct in treatments where warming and nutrient loadings were coupled and fish losses were attributed to frequent, severe hypoxic episodes. At an ecosystem level, eutrophication in lakes can lower ecological specialization and promote genetic and phenotypic homogenization [50], but it remains unknown if these effects hold with climate warming. Models based on field data suggest that nutrient pollution and high temperatures in combination will drive population declines in macroinvertebrates and fish at regional scales [51]. Scaling up interactions between eutrophication and warming from species- to population-level effects should be a priority for future investigations.

## 5. New directions and conclusion

Conserving and managing aquatic ectotherms is becoming increasingly challenging as climate change interacts with threatening processes. Investigations have primarily focused on understanding how climate warming interacts with the process of eutrophication (e.g. algal bloom formation and sediment loads), rather than understanding how aquatic ectotherms are affected by the combination of these threats. Current understanding suggests that chronic exposure to particular nutrients can increase fish susceptibility to acute temperature spikes and hypoxia [29,30,46], but research is needed on a greater diversity of species to test the wider applicability of this interaction. Nonetheless, these data suggest that nutrient pollution is not only causing a range of sub-lethal effects on aquatic ectotherms [28], but is also increasing their vulnerability to climate change.

Available evidence also suggests that gradual warming and heatwaves have divergent interactions with nutrient pollution. Thermal acclimation responses, associated with gradual warming, induce physiological changes in aquatic ectotherms (e.g. gill, ventricle and haematological remodelling) that offer protection against nutrients and hypoxia [40–42]. By contrast, acute increases in temperature increase the susceptibility of many aquatic ectotherms to hypoxia [43]. Therefore, gradual climate warming may aid aquatic ectotherms in coping with eutrophication, but heatwaves pose a threat.

Well-designed, mechanistically focused studies offer a fruitful approach to elucidating *cause and effect* behind interacting threats in a changing world. Only full-factorial experimental designs, which examine both the isolated and combined effects of threats, can effectively disentangle interactions. Mimicking threats in ways that enhance ecological realism is vital. Most studies have only assessed two-way threat interactions, and very little is known about how three or more threats interact, despite eutrophication involving a mixture of threats. Moreover, multi-threat studies generally expose organisms to threats simultaneously, but this approach does not always reflect natural processes, with some threats experienced sequentially in nature (figure 1*b*). Manipulating the order that organisms are exposed to threats can reveal insightful cross-tolerance or cross-susceptibility interactions, which may inform management practices [52]. Management practices often operate to remove threats; for example, restricting nutrient loads in habitats. Therefore, we also need to understand how species respond to the removal of a threat and track the recovery of populations.

Better mimicking climate change scenarios in experiments will also enhance ecological relevance. Ectotherms are projected to be more vulnerable to increases in temperature variability than increases in mean temperatures [53]. Despite this, we lack an understanding of how increased thermal variability interacts with eutrophication because studies have only used stable, constant temperatures. Mesocosm experiments [54] and ecological/mechanistic niche modelling [55] will be paramount to testing if mechanisms identified in tightly controlled experiments scale up to impact population and community level dynamics under increasingly natural conditions. Current research suggests there is a looming threat of combined climate warming and eutrophication for aquatic ectotherms, but the impacts of gradual warming and heatwaves will likely be distinct. Further mechanistic research on taxonomically diverse species is required to deepen our understanding of these interactions and to develop management solutions.

## Data accessibility

This article does not contain any additional data.
